# Saliva detection of SARS-CoV-2 for mitigating company outbreaks: a surveillance experience, Milan, Italy, March 2021

**DOI:** 10.1017/S0950268821001473

**Published:** 2021-07-30

**Authors:** Emerenziana Ottaviano, Chiara Parodi, Elisa Borghi, Valentina Massa, Cristina Gervasini, Stefano Centanni, Gianvincenzo Zuccotti, Silvia Bianchi, Silvia Ancona, Elisa Adele Colombo, Elisabetta Di Fede, Paolo Grazioli, Elena Lesma, Antonella Lettieri

**Affiliations:** 1 Department of Health Sciences, Università degli Studi di Milano, Via Rudinì, 8, 20142 Milan, Italy; 2 Department of Paediatrics, Children Hospital V. Buzzi, Università degli Studi di Milano, Milan, Italy

**Keywords:** Control measures, COVID-19, saliva sample, SARS-CoV-2, surveillance

## Abstract

Monitoring the severe acute respiratory syndrome coronavirus 2 (SARS-CoV-2) community-wide transmission with a suitable and effective sampling method would be of great support for public health response to the spreading due to asymptomatic subjects in the community.

Here, we describe how using saliva samples for SARS-CoV-2 detection has allowed for a weekly surveillance of a small business company and the early detection of coronavirus disease 2019 cases.

As on 23rd March, two cases were detected and investigated, and control measures were rapidly applied.

At the beginning of November 2020, a small business company implemented a weekly surveillance of all employees based on molecular detection of severe acute respiratory syndrome coronavirus 2 (SARS-CoV-2) infection using saliva as sampling method (planned surveillance). Active surveillance in closed communities based on multiple saliva testing could be a comprehensive approach to identify asymptomatic subjects and to reduce the transmission of SARS-CoV-2 infection [1, 2]. Saliva specimens allow for self-collection and repetitive testing by reducing the discomfort, thus increasing subject compliance [3, 4]. Hence, this strategy was envisaged for an elevated compliance and attendance to the surveillance activity. All 32 employees located in the surrounding area of Milan agreed to participate in such surveillance. The small business company consists of 10 different open space offices (three to six people per office, depending on the office size) located on two different floors, and one office with six employees in a nearby building. In the main building two kitchen areas for coffee breaks (maximum three people at one time) and a lunchroom (maximum six people at one time) were accessible for all the employees.

Employees were asked to provide a self-sampled saliva and a self-evaluation form with information on body temperature and presence of commonly referred coronavirus disease 2019 (COVID-19) symptoms.

COVID-19 cases were defined by a positive SARS-CoV-2 test. SARS-CoV-2 laboratory confirmed cases and their close contacts were tested every three days from the first positive SARS-CoV-2 test for 14 days (tight-mode).

Self-sampled saliva was collected using an *ad hoc* designed disposable prototype (LolliSponge™, Copan, Brescia, Italy) consisting of sponges inserted on a plastic shaft, fixed on a screw cap. A plastic tube allows to protect collected samples during transport to the laboratory. This system was validated by comparing paired nasopharyngeal swab (NPS) and LolliSponge™ samples (Supplementary Fig. S1).

Briefly, the LolliSponge™ was kept for one minute in the mouth allowing for the sponge to soak true saliva. The device is conceived so that the sponge does not come in contact with hands whereas can be safely stored in a collecting tube. Samples, maintained at room temperature (RT) and without transport medium as saliva is self-preservative, were delivered and tested for SARS-CoV-2 by quantitative reverse transcription polymerase chain reaction (qRT-PCR) assay at the microbiology laboratory of the Department of Health Sciences of the University of Milan, as previously described [5] with few adaptations. Saliva was recovered by centrifuging the device for 1 min at 500 × g. Briefly, 5 μl of saliva, upon treatment with proteinase K and heat inactivation, were used for RT-qPCR for the simultaneous detection of N1 (FAM probe), N2 (SUN) and human RNase P (RP, ATTO647) genes using the protocol published by the Centers for Disease Control [6]. Samples were considered positive upon detection of N1 and/or N2 (Ct <40). Invalid samples were assessed by RP (Ct >35).

A total of 32 subjects (mean age 39.5 years, range 25–71), 22 females and 10 males, underwent weekly testing. According to National laws [7], 30%–70% of staff was allowed to work in the office, leading to a variation in the planned frequency of sampling.

Starting from the 6th of November, a total of 397 samples were analysed, with a mean of 12.41 samples/persons during the surveillance time and 2.5 samples/month/persons.

On the 17th of February, the first positive case was detected (N1 Ct = 34.8, N2 Ct = Undetected, RP Ct = 27.5). The subject (C1) was a healthy 38-year-old female and was promptly isolated and contact tracing started. Because of the National Law, the subject was attending work fortnightly, and did not report any direct contact with colleagues or family members for the previous seven days. She recounted a meeting with few friends that were later found positive by National Health Service (NHS) NPS testing. The planned tight-mode surveillance did not highlight any other positive results. C1 was always asymptomatic.

On the 5^th^ of March another case (C2), a healthy 71-year-old man, tested positive (N1 Ct = 20.5, N2 Ct = 25.5, RP = Ct 26.6) triggering contact tracing and tight-mode surveillance (work and household contacts of positive cases were offered testing every 3 days). The day after, his family member (C2.1), a previously healthy 71-year-old woman, also tested positive, and developed fever. Both subjects presented with interstitial lung disease (ILD) on the 15th of March, about 10 days after their positivity in saliva.

In the company, five people were considered at risk for sharing open space and three for sharing the lunch break. All these close contacts tested negative for SARS-CoV-2 on the 5th of March. They were all advised to increase protective measures at home. On the 8th of March, two (C2.2 and C2.3) of the eight contacts became positive to SARS-CoV-2 infection, all of whom shared the open space office with the index case. C2.2, a healthy 42-year-old man, presented mild symptoms and remained positive for 11 days. He was isolated from his family on the 6th of March and none of his four family members showed positive results. C2.3, a healthy 43-year-old woman, did not implement any isolation measures at home and both her family members (C2.3.1 and C2.3.2) tested positive in saliva on day 6. Four days after the detection in saliva, C2.3 and her family members presented COVID-19 symptoms. At the end of the 14 days-tight surveillance none of the 29 other employees was infected (Fig. 1). To note, hospitalised cases in our study resulted from a sustained exposure (at home).

**Fig. 1.**
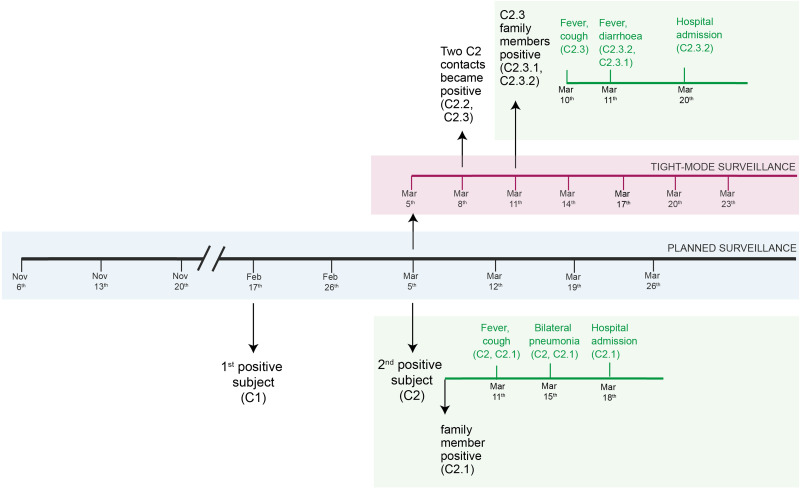
Timeline of the active surveillance: in grey, planned surveillance; in purple, tight-mode surveillance; in green, self-reported clinical data.

In this report, we present the possible advantage of an active surveillance using saliva samples for the early detection of SARS-CoV-2 [8] and the mitigating effects on COVID-19 outbreak in a small business company in the metropolitan area of Milan, Lombardy, one of the areas most affected by SARS-CoV-2 pandemic in Italy. In our study, positive cases were detected during the two peaks of viral circulation in Lombardy (November 2020 and March 2021) [9]. Given that, in our country, mainly symptomatic subjects are normally tested, the percentage of positive subjects in our asymptomatic cohort is difficult to be compared with officially reported numbers.

Further, it is possible to hypothesise a higher compliance to suggested mitigation measures – i.e., masking, distancing, air changing etc. – in a population actively seeking for surveillance.

Viral replication in saliva starts in the very early phases of SARS-CoV-2 infection, and viral load is independent of symptom severity [10], thus permitting to identify infectious subjects timely, also in asymptomatic individuals [11]. Clearly, saliva droplets represent a too often underestimated but extremely efficient route of transmission, as described also for other respiratory viruses [12].

This study exemplifies how measures based on case-based surveillance − i.e., of notified cases, mostly symptomatic [13] − fail to control or mitigate viral spread. All the positive subjects identified by the planned surveillance were asymptomatic at the time of testing, with no recognised risk of exposure.

Based on our data, whereby implementing recommended control measures led to interrupting the outbreak [14], we suggest designing active surveillance based on saliva for closed communities, even on a bigger scale such as schools and residential care home. Timing could be fine-tuned according to local epidemiological situation and tightened upon positive cases. Larger studies evaluating costs, logistics and feasibility are currently undergoing in our country.

## Supplementary material

For supplementary material accompanying this paper visit http://dx.doi.org/10.1017/S0950268821001473. click here to view supplementary material

## Data

The data presented in this study are available on request from the corresponding author.
