# Supplementary material for: Saliva detection of SARS-CoV-2 for mitigating company outbreaks: a surveillance experience, Milan, Italy, March 2021
Source: Epidemiol Infect. 2021 Jul 30;149:e171. doi: 10.1017/S0950268821001473 (PMC8365045; doi:10.1017/S0950268821001473)

*Epidemiology and Infection*

**Saliva detection of SARS-CoV-2 for mitigating company outbreaks: a surveillance experience, Milan, Italy, March 2021.**

Emerenziana Ottaviano, Chiara Parodi, Elisa Borghi, Valentina Massa, Cristina Gervasini, Stefano Centanni, Gianvincenzo Zuccotti, Lollipop Study Group, Silvia Bianchi.

**Supplementary Material**


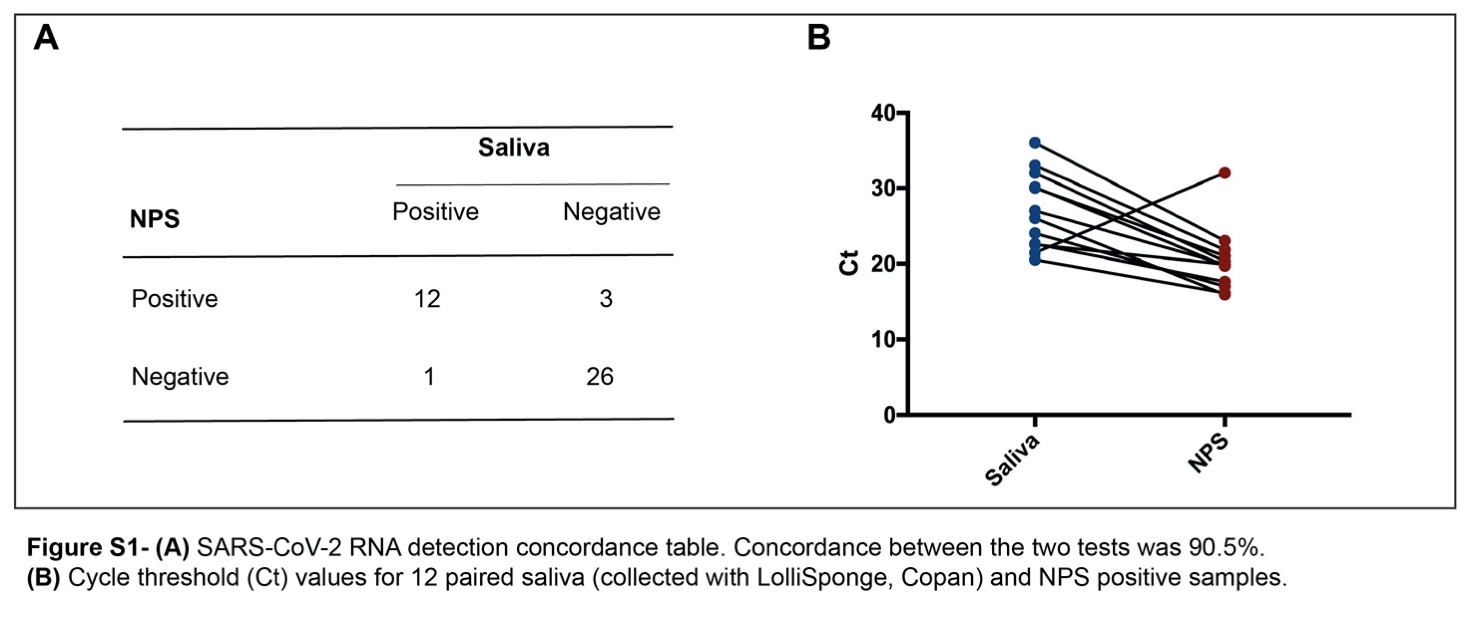

Supplement: Supplementary file 1 [file S0950268821001473sup001.docx]
